# Regulatory framework for dietary supplements and the public health challenge

**DOI:** 10.11606/s1518-8787.2019053001263

**Published:** 2019-10-16

**Authors:** Thaís Ramos Dal Molin, Gabriela Camera Leal, Larissa Sabo Müller, Diana Tomazzi Muratt, Gabriela Zanella Marcon, Leandro Machado de Carvalho, Carine Viana

**Affiliations:** I Universidade Federal de Santa Maria. Programa de Pós-Graduação em Ciências Farmacêuticas. Santa Maria, RS, Brasil; II Universidade Federal de Santa Maria. Programa de Pós-Graduação em Química. Santa Maria, RS, Brasil; III Universidade Federal de Santa Maria. Departamento de Química. Santa Maria, RS, Brasil; IV Universidade Federal de Santa Maria. Departamento de Farmácia Industrial. Santa Maria, RS, Brasil

## Abstract

**OBJECTIVE:**

The new regulatory framework for dietary supplements in Brazil prompted this analysis of the current outlook of these products and the challenges posed by the new guidelines.

**METHODS:**

We conducted a qualitative, observational and descriptive study of dietary supplements commercialized in Brazilian online stores with the help of the Google® search tool. We considered the ingredients on the labels, the effects attributed to these products and the commercial claims used as a means of promoting them to assess the necessary changes for the legal framework in the new guidelines. Finally, with the help of a database, we compared the effects declared by the manufacturers and attributed to certain ingredients with the scientific evidence described in literature.

**RESULTS:**

In total, we purchased 44 dietary supplements from Brazilian online stores (n = 7). Of the samples studied, 34.2% could not be classified in the category Dietary Supplements, as recommended by the new regulation of the Brazilian Health Regulatory Agency due to the presence of prohibited substances; 16% of products should be commercialized as medicines. Regarding the commercial appeals, 97.7% had banned expressions. Numerous claims of effects attributed to certain products were characterized as consumer fraud because they have no scientific evidence.

**CONCLUSIONS:**

The necessary changes represent a major regulatory and production challenge due to the wide range of dietary supplements and markets, an effort that aims to protect the consumers’ health. Some previous gaps in the regulatory framework were not fully solved.

## INTRODUCTION

The consumption of dietary supplements has been increasing significantly in recent years and numerous reasons justify the phenomenon. Easy purchase in e-commerce and the social media’s strong influence are among the biggest impact factors. From 2010 to 2016, the consumption of dietary supplements in Brazil increased 233%, reaching a turnover of 1.49 billion reais^1^ . The large number of people consuming these products on their own initiative or through friend’s advice without the proper recommendation of a specialized professional has become a public health problem, worrying experts and authorities^2^ . Literature reports numerous undesirable adverse effects, liver damage and even cases of death due to ingestion of dietary supplements^3^ .

In Brazil, until 2018 dietary supplements had no legal definitions. According to the Brazilian Health Regulatory Agency (ANVISA ), most products used as dietary supplements were classified into different regulatory categories: (I) Food for athletes; (II) Vitamin and/or mineral supplements; (III) New foods and/or new ingredients; (IV) Food of functional and/or health property; (V) Specific medications; and (VI) Herbal medicines^6^ . The large number of products on the market makes inspection difficult, and the lack of safety for consumer health is the biggest risk associated with this outlook. This scenario favors the commercialization of dietary supplements containing proscribed substances in Brazil, such as ephedrine and 1,3-dimethylamylamine^12^ . Cases of dietary supplements deliberately adulterated to intensify the pharmacological effect and promote a false image that the product works are widely described in literature^3 , 5 , 13^ . In addition, the lack of official methodologies in Brazil for this type of analysis makes the quality and safety of these products questionable. Consumer fraud was also connected to dietary supplements commercialized in Brazil due to divergences between the amount of carbohydrate and protein declared on labels and the actual values found after analysis by ANVISA^16^ .

In recent years, ANVISA held several meetings to discuss and define regulation for dietary supplements. These debates originated the ANVISA RDC No. 243/2018, which defines the sanitary requirements for dietary supplements and is characterized as a regulatory framework in Brazil^17^ . ANVISA also established the food additives and supporting technologies authorized for use in dietary supplements through ANVISA RDC No. 239 of July 26, 2018^18^ . In contrast, the reference daily intake (RDI) values for vitamin, mineral and amino acid products are no longer the differentiating factor between vitamin and/or mineral supplements and specific medicines^7 , 10^ . According to the new ANVISA RDC No. 242/2018, only some products will be considered specific medicines: the vitamins and/or minerals and/or amino acids and/or protein based ones, isolated or associated with each other, for oral use, with well-established and therapeutic indications and different from the claims used in dietary supplements^19^ .

The regularization of commercially available products under the new legal guidelines ranges from changes in label information to changes in the composition of dietary supplements. The regulated sector has five years to adapt to the new resolutions.

This study evaluated the main non-compliances observed on dietary supplement labels and verified whether the effects attributed to some ingredients had scientific evidence support. We also indicated the changes required by the new legislation for the most popular products commercialized in online stores.

## METHODS

### Acquisition of samples

We conducted a retrospective, observational and descriptive study of the information collected from the purchase moment until the delivery of dietary supplement samples. We purchased the samples from January to December 2017, before the regulatory framework for dietary supplements, and analyzed them according to the valid legislation at the time for this category of products. We also assessed non-compliance occurrences and required changes for the legal framework of the samples in the current guidelines. We considered relevant all the products that presented “food for athletes” or “dietary supplement for physically active people” as indication. We acquired the samples online, searched with the help of the Google® search tool. The e-commerce stores (n = 7) were all Brazilian, with *Cadastro Nacional de Pessoa Jurídica* (CNPJ – National Registry of Legal Entities), that declared the physical address on their websites and provided customer service. Sample prices ranged from R$ 19.50 to R$ 244.45. According to the available budget and the characteristics required to frame the product as a sample of interest, we could acquire 44 samples, which we received via mail.

### Sample Classification

The dietary supplements were distributed into categories designated according to ratings given by the websites (hormone modulation supplements, weight loss supplements, sexual performance supplements and muscle builders). The pertinent labeling information, such as ingredients, batch, manufacturer, images or expressions, information on exemption of sanitary registration according to ANVISA RDC No 27/2010^20^ and indication for consumption, among others, were tabulated with the help of Microsoft Excel®. We assessed these data for compliance according to the valid legislation at the time of acquisition^6^ . Subsequently, we analyzed the samples based on the new classification criteria for dietary supplements, permitted ingredients and claims referred in ANVISA RDC No. 242 and 243/2018^17 , 19^ and attachments. The claims attributed to the major ingredients of samples were compared with the recommendations of international societies and official organizations, such as the International Society of Sports Nutrition^21^ , Academy of Nutrition and Dietetics, Dietitians of Canada and American College of Sports Medicine^22^ , as well as scientific articles.

## RESULTS

We named the effects that the e-commerce sites attributed to products as “commercial appeal.” According to the commercial appeals on the websites, the samples were classified into four main categories: (I) Hormonal Modulator/Precursor; (II) Weight Loss/Appetite Control/Diuresis; (III) Increased libido/sexual potency; and (IV) Muscle Builder/Performance Enhancer/Strength Gain. Chart 1 shows the frequency of these categories, as well as the effects attributed by manufacturers as a form of product advertising. The presence of more than one declared “active” ingredient on the label of some products was one of the main points we observed, which made the legal framework difficult in some legislations. Chart 1 shows only the major ingredients, highlighting whey protein, natural ingredients (plant species) and vitamins and/or minerals as the most frequent.

**Chart 1:** Frequency among product categories, effects attributed to the products observed on the label and the major ingredients of the studied dietary supplements.

Category % (n)			Effects attributed to products		Main Ingredients	

					Description	n
I	Hormonal modulator/precursor	29.6 (13)	Precursor/synthesis of/ enhancer/increase of	Testosterone	Zinc, Magnesium and Vitamin B6^a^	7
					DHEA^b^	2
					*Tribullus terrestris*	2
					Peruvian ginseng	1
					Chromium picolinate	1
II	Weight Loss/Appetite Control/Diuresis	18.2 (8)	Moderator	Appetite	Caffeine	3
					Vitamins and/or minerals	3
					*Citrus aurantium*	1
					Psyllium ( *Plantago ovata* )	1
			Enhances/Increases	Diuresis		
			Assists/promotes	Weight loss		
III	Increases / strengthens and combat / treatment of sexual potency/libido	13.6 (6)	Increase/Enhance	Virility and libido	*Tribullus terrestris*	2
					Peruvian ginseng	2
					*Eurycoma longifolia*	1
					Zinc, Magnesium and Vitamin B6	1
			Combat/treatment of	Sexual impotence		
IV	Assist / increase/ define and promote/ provides muscle Builder/Performance Improvement/Strength Gain	38.6 (17)	Assist/increase/shape	Muscle Mass	Whey Protein	7
					BCAA^c^	3
					*Paullinia cupana*	2
					Maltodextrin	2
					Zinc, Magnesium and Vitamin B6	2
					Beef Protein	1
			Promotes/Provides	Anabolic Effects		

		100 (44)			Total	44

^a^ Ingredient declared on the label in two ways: I) under the name ZMA; II) as nutrients added in different proportions.

^b^ Dehydroepiandrosterone.

^c^ Branched-chain amino acids.

According to the manufacturers, samples declared as hormone modulators (category I) (n = 13) were able to increase testosterone production or suggest the presence of precursor ingredients of this hormone. Six samples contained plant species prohibited in dietary supplements, such *as Tribullus terrestris* and *Paullinia cupana* , claiming to have herbal properties according to ANVISA RDC No. 26/2014^11^ and the Brazilian pharmacopeia National Form^23^ . Currently, according to the Normative Instruction (NI) ANVISA No. 28 of July 26, 2018, *Paullinia cupana* is among the authorized constituents of dietary supplements as a caffeine source^24^ . In the same category, two samples contained dehydroepiandrosterone (DHEA), a medicine whose use is controlled in Brazil according to Ordinance MS No.344/1998^25^ .

Among the samples alluding to weight loss (category II) (n = 8), all had commercial pharmacological action appeals such as “appetite suppressant” or “increased diuresis.” One of the products that claimed to be exempted from registration as “Athlete Food” could not be legally classified in this category, as it declared *Citrus aurantium* on the label, a plant species that classifies the product as an anxiolytic herbal medicine. Its administration is recommended only for those over 12 years old, which therefore requires ANVISA registration for its commercialization^11 , 23^ .

All supplements indicated to increase libido (category III) (n = 6) were in disagreement with the legislation as they attributed therapeutic effects to the products. Four of the supplements declared the presence of plant species, among them the herbal medicine *Citrus aurantium* ^11 , 23^ , *Tribullus terrestris* and *Eurycoma longifolia* . The last two are not available on the Herbal Medicines Form but are susceptible to registration due to the therapeutic claims presented^26^ .

Supplements with muscle building claims or related to physical performance were the most frequent (category IV) (n = 17). Ten products were correctly classified as “Athlete Food,” considering the ingredients stated on the labels, which were exempt from registration^6 , 20^ . Seven products had advertising alluding to anabolism, which is not allowed in food products^6 , 27^ , for example, supplements containing only vitamins and/or minerals with claims such as: “Generates muscle mass and strength” or “Promotes muscle growth.” Two samples declared exemption from registration as they were called “Athlete Food”; However, due to the presence of substances that have no record of consumption in the country, such as the Peruvian ginseng ( *Lepidium meyenii* ), they should fall under the category “New foods and/or new ingredients”^8 , 20^ . According to the legislation in force at the time of purchase of dietary supplements, two samples had non-nutrients or dietary fiber added, which was prohibited, as per item IV of article 8 of ANVISA RDC No.18/2010^6^ . Under the new guidelines, this addition is possible as long as the substances are on the list of authorized constituents and the minimum, maximum limits and assigned claims are respected^17 , 24^ .

**Figure 1:**
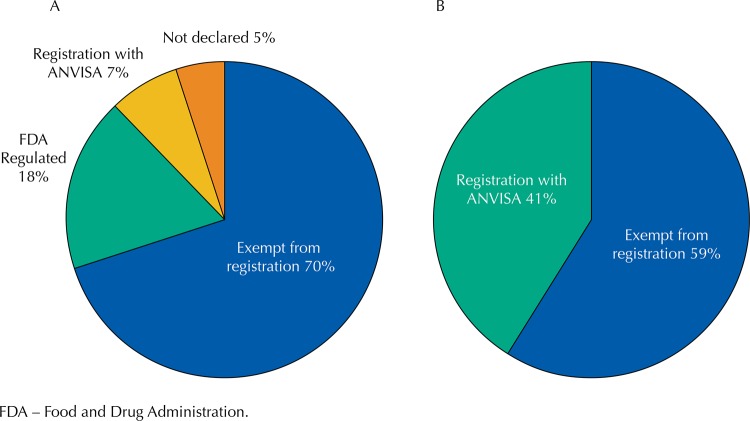
Graphical representation of the evaluated sample records: (A) manufacturer declarations; (B) ideal declarations, in accordance with valid regulatory guidelines.

Concerning the necessity of registration of samples with ANVISA, for both national and international products (n = 4), we performed a comparative analysis between what manufacturers stated on labels ( Figure 1A ) and the ideal declaration according to legislation in force at the time of production ( Figure 1B )^20^ .

FDA – Food and Drug Administration.

**Figure 2:**
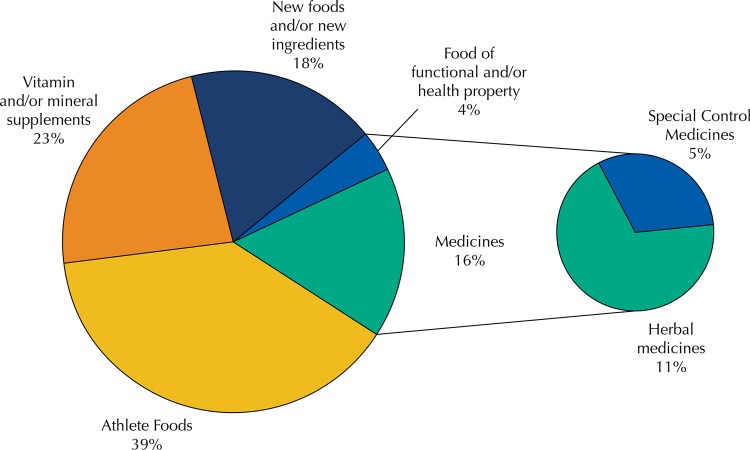
Distribution of samples evaluated according to regulatory categories, according to the legislation in force at the time of product acquisition.

Figure 2 shows the discrepancy of difference between the regulatory framework of products according to the information stated on the label ( Figure 1A ) and what the distribution between categories should be after analysis of declared ingredients. Among the samples that erroneously claimed to be exempt from registration were new foods, food of functional property and even medicines.

According to the new regulation, most products analyzed (70.5%, n = 29) were in compliance with the list of ingredients allowed in dietary supplements^17 , 24^ . Some ingredients ( *Paullinia cupana* and other non-nutrients in athlete food, for instance) that, until the time of the sample acquisition, were not legally protected to be added, are now allowed to be part of the dietary supplement constituents, respecting the necessary requirements^17 , 24^ . On the other hand, 15 samples (34.2%) could not be commercialized as dietary supplements because they contain ingredients that are missing from the list of authorized constituents, such as *Camellia sinensis, Lepidium meyenii,* ginger, cinnamon powder, *Trichilia catigua* . Regarding the label, 97.7% of the samples were not in conformity due to the use of unauthorized expressions in dietary supplements, which attributed medicinal or therapeutic properties to the product, such as increased libido and diuretic action^17 , 27^ . In addition, we also observed the use of images alluding to pharmacological effects such as weight loss or improved sexual performance^17^ .

## DISCUSSION

### Label Analysis

Until 2018, the name “dietary supplement” was not provided by law and any product for this purpose was commercialized as food in several categories. We acquired the study samples before the Brazilian regulatory framework that defined the dietary supplements and whose main objective was to reduce regulatory fragmentation. Until then, sanitary registration was mandatory for “New foods,” “Foods with functional properties” and “Foods containing bioactive or probiotic substances,” while “Athlete foods” and “Vitamin and/or mineral supplements” were exempt from registration^20^ .

The non-conformities found in the study samples were: (I) absence of product registration in the categories “New foods” (n = 8) and “Foods with functional property” (n = 1); (II) presence of ingredients that characterized the products as medicines (n = 7); (III) use of improper expressions and allegations (n = 43).

DHEA, for example, declared in two samples, is prohibited in dietary supplements commercialized in Brazil, as it is an anabolic steroid precursor, susceptible to special control^25^ . By contrast, in the United States, this medicine is authorized in dietary supplements. However, numerous serious adverse effects are related to its consumption, such as tachycardia, arrhythmias, hepatotoxicity, among others^28^ . In addition, five samples declared the presence of unauthorized plant species such as *Tribullus terrestris* and *Citrus aurantium* . However, only *Citrus aurantium* has safety and efficacy based on clinical evidence, characteristics attributed to herbal medicines^11^ . The presence of banned substances in dietary supplements can cause serious public health problems. The user believes that it is a “natural product” and, therefore, will not harm health, which promotes excessive and often unnecessary consumption and may cause health damage, such as hepatotoxicity, cardiotoxicity and even death^5 , 29^ .

Products commercialized as “Athlete Foods” had restrictions on the composition and addition of other substances, according to the intended use. Protein supplements, for example, conforming to previous legislation, could not contain fibers and non-nutrients. Caffeine supplements could not contain any nutrient or non-nutrient added in the formulation^6^ . Due to the legislation in force at the time of samples acquisition, eight of them presented this type of non-compliance.

In Brazil, the addition of herbal medicines to athlete foods is prohibited^11^ . We observed this non-compliance in a sample containing caffeine associated with *Citrus aurantium* . High caffeine concentrations may promote toxic effects, such as nausea, vomiting, arrhythmias, tachycardia, and may even lead to death^30^ . Synephrine is one of the active compounds in *Citrus aurantium,* often found in weight loss products. However, studies indicate possible cardiotoxicity related to indiscriminate consumption^31^ . In Canada, for example, caffeine or caffeine sources addition to synephrine-containing products is banned due to the synergism of action, which may promote cardiotoxic effects, thus requiring proven safety evidence in humans^32^ .

These label non-conformities serve as primary tools for assessing the quality of the product on the market. The inspection by the responsible bodies is fragile, probably due to the large number of available dietary supplements and stores, as well as the ease of purchase enabled by e-commerce. The difficulty of legal framing of the products by manufacturers, sellers and consumers was evident because of fragmented and unspecific regulation in force during the samples acquisition.

### Commercial Appeals of Products

Commercial appeals are advertising tools designed to draw attention to possible benefits that the formulation or some specific component promotes in the body. Since its implementation, ANVISA RDC Resolution No. 259/2002 states that foods, regardless of category, cannot have indications of medicinal or therapeutic properties^27^ . Expressions that refer to anabolism, catabolism, hypertrophy, fat burning, or increased sexual ability are vetoed on labels, advertisements, pamphlets, or other commercial tools for products classified as athlete food^6^ and are currently considered dietary supplements^17^ . This non-compliance is susceptible to penalty, such as suspension of the product sale, fine and even website exclusion. In this study, we observed a significant number of samples with commercial appeals and misuse of expressions (97.7%). We suggest the following hypotheses: (I) High number of websites; (II) Difficulty to inspect online stores; (III) The exclusion of an inappropriate site is not a hindrance to the opening of another; (IV) Manufacturers are not always the ones who make the commercial appeals for the products, but the distributors, to increase sales. Using sentences that induce consumers to buy can be considered consumer fraud, as it is not always possible to guarantee that a particular ingredient will provide the expected result.

### Effects Attributed to Main Ingredients and Their Scientific Evidence

Numerous substances are present in dietary supplements, but their presence is not always justified in consolidated scientific evidence. The effects attributed to the ingredients of these products are often not substantiated and sometimes inconsistent with those already described. Gaps in inspection contribute to the high number of products that deceive consumers with their miraculous effects, prompting them to buy. The regulatory framework emerged to attempt to reduce these gaps through a list of permitted claims related to the constituents of dietary supplements available in Attachment A of ANVISA Regulatory Instruction No. 28 of July 26, 2018^24^ . The frequent presence of ingredients from plants, vitamins and minerals in the samples and the image of “natural product” they convey is also relevant. The consumer generally believes that, for being a natural product, it is safe and will not harm the body^13 , 14^ .

***According to Rocha et al. (2016)^3^ , the great demand for these products is due to three factors: (I) growing distrust in conventional medicine, with a greater interest in alternative therapies; (II) the perception that they are “natural,” “healthy” products and safe, for being plant products; and (III) a growing trend of self-medication as a way to increase control over one’s health.

The addition of plants is directly related to the indication to reduce measures, diuresis or other claims that induce weight loss. However, a species may contain more than one chemical, and an incorrect plant extraction may isolate unwanted metabolites. Thus, the addition of medicinal plants should be carefully observed.

Tribullus *terrestris,* declared in four samples, is a plant extract that has been connected to luteinizing hormone (LH) stimulation and consequent increase in testosterone^33^ . Studies suggest that due to the presence of protodioscin, it has a steroid-like activity. However, no evidence shows that Tribullus *terrestris or* any single compound of this species promotes significant changes in body composition, such as lean mass gain or anabolism^33^ .

Caffeine is a natural substance (1,3,7-trimethylxanthine) and widely consumed worldwide as a stimulant in dietary supplements, especially those indicated for “natural” weight loss. This methylxanthine is added with the commercial appeal of accelerating metabolism, stimulating diuresis and promoting fat burning. However, official positions detach caffeine from an increase in fat oxidation rate, but attribute caffeine to an increase in athlete performance as a potent brain stimulant^21 , 22^ .

Protein foods for athletes have become popular among gym-goers for their association with lean mass gain. Whey protein is nothing more than the supernatant obtained from casein coagulation, accounting for 15-20% of total milk. The new regulation claims that “proteins help in the formation of muscles and bones”^24^ . Protein supplements may be one of the few in accordance with scientific evidence for lean mass gain, as they help in muscle building. However, they are dissociated from testosterone levels or other ergogenic effects^21 , 22 , 34^ . Claims found in seven samples, which used expressions referring to whey protein as capable of “increasing hormone levels” or as “an important precursor of testosterone.”

The branched-chain amino acids (BCAA), declared in three samples, also conquered their place inside the gyms. Consisting of leucine, isoleucine and valine, BCAA are consumed to reduce both physical and mental fatigue, improve performance and promote ergogenic effects such as anabolism. BCAA are responsible for tryptophan uptake and its conversion to serotonin, which is responsible for muscle fatigue^35^ . However, the beneficial effects of this type of supplement are still divergent in the literature.

In Brazil, BCAA supplements, until the moment of acquisition of the samples, could not state indications for athletes or as athlete food, even with registration exemption^6^ . However, we observed this non-compliance in all BCAA or BCAA-containing supplements (6.8%), as they were purchased with the athlete food specification.

Vitamins and minerals are dietary supplements intended to supplement the dietary needs of a healthy person, without the intention of replacing any food or being used as an exclusive diet^7 , 19^ . At the time of the acquisition of samples, ANVISA’s regulation considered that vitamins and minerals were foods when they contained 25 to 100% of the reference daily intake (RDI) in the portion indicated on the label^7^ . If the product contained doses with concentrations equivalent to more than 100% of the RDI, it was considered a specific medicine^10^ . Currently, the new regulation considers vitamins and minerals as dietary supplements, regardless of the dose. They will be considered specific medicines only when they have well-established therapeutic claims, such as ferrous sulfate, which helps in deficiency anemias, and folic acid, which is indicated for reducing the occurrence of fetal malformations^19^ .

Recently, the consumption of dietary supplements containing zinc and magnesium, especially among men, became popular due to the belief that this combination could increase testosterone levels and thus promote anabolic effects^36^ . These minerals increase insulin-like growth factor (IGF-1) concentrations in children with disabilities; However, this claim has no proven evidence in healthy people^37 , 38^ . We found claims in three samples that the combination of zinc, magnesium and vitamin B6, also known as ZMA, contained testosterone precursor substances. No scientific evidence shows that this supplement aids in training adaptations, promotes anabolic effects, or promotes significant effects on testosterone levels or metabolism^21 , 39^ .

The miraculous effect attributions to substances in dietary supplements, or even designations of “magic formulations,” are recurrent in these products. Manufacturers are not always the ones who attribute those claims, but rather shop owners as an advertising to boost sales. These miraculous attributions can also be an indication of the presence of prohibited substances in foods. The case of OxyElite Pro®, for example, which was widely disseminated because of its action on weight loss, contained 1,3-dimethylamylamine (DMAA), a highly harmful substance to the body. From February 2012 to February 2014, 114 cases were reported to the Food and Drug Administration (FDA) of adverse effects related to the consumption of this supplement, characterizing a serious public health problem^5 , 40^ .

### Regularization of Products Available in the Market

The new legislation for Brazilian dietary supplements seeks to ensure quality consumer products with less regulatory hurdles for manufacturers. The products evaluated in this study need to undergo several changes to fit the new guidelines. These modifications range from composition (since current regulations provide a list of permitted ingredients and additives) to labeling regularization and claims used to promote the product^17^ .

We observed a high frequency of advertisements about certain ingredients, which are not always true. These commercial appeals are intended to induce the consumer to buy or promote the false image that the product works. Regarding the allegations, according to the new legislation, of the 44 samples analyzed, only two would be in accordance with the rules set in Annex V of Normative Instruction No. 28, of July 26, 2018^17 , 24^ . Advertising rules are less complied by manufacturers and distributors due to the difficulty of regulatory agency inspection. It is often more advantageous for manufacturers to pay fines due to appealing claims as they boost sales and popularization of the product. The current regulatory system for dietary supplements aimed to reduce these non-compliances through the list of authorized constituents, their minimum and maximum limits and permitted claims. However, the new guidelines are not yet enabling tools for both industries and consumers because of some markers. For example, the manufacturer will define the appropriate quantities for daily consumption recommendations of the product and by population group in cases where minimum and maximum limits are not established^17^ .

The growth in the number of dietary supplements available on the market, as well as the high number of stores, contributes to the inefficiency of the inspection of these products. The ease of purchase via e-commerce can make consumers have access to products that are illegal or that contain prohibited substances in dietary supplements. The user is not always aware of the damage these substances can cause, posing a public health challenge. Education policies that show the risks associated with unnecessary consumption, warnings that help to recognize the quality of dietary supplements, and research for a skilled professional before purchasing any of such products are good strategies for raising awareness and therefore reducing sales of illegal products or those that violate the law.
