# Supplementary material for: Regulatory framework for dietary supplements and the public health challenge
Source: Rev Saude Publica. 2019 Oct 16;53:90. doi: 10.11606/s1518-8787.2019053001263 (PMC6802945; doi:10.11606/s1518-8787.2019053001263)
Supplement: Translation

# Translation: Marco regulatório dos suplementos alimentares e o desafio à saúde pública

**Authors:** Thaís Ramos Dal Molin, Gabriela Camera Leal, Larissa Sabo Müller, Diana Tomazzi Muratt, Gabriela Zanella Marcon, Leandro Machado de Carvalho, Carine Viana

**Affiliations:** I Universidade Federal de Santa Maria. Programa de Pós-Graduação em Ciências Farmacêuticas. Santa Maria, RS, Brasil; II Universidade Federal de Santa Maria. Programa de Pós-Graduação em Química. Santa Maria, RS, Brasil; III Universidade Federal de Santa Maria. Departamento de Química. Santa Maria, RS, Brasil; IV Universidade Federal de Santa Maria. Departamento de Farmácia Industrial. Santa Maria, RS, Brasil

**Keywords:** Suplementos Nutricionais, Comercialização de Produtos, Qualidade de Produtos para o Consumidor, legislação & jurisprudência, Agência Nacional de Vigilância Sanitária

## Abstract

**OBJETIVO:**

O novo marco regulatório para os suplementos alimentares no Brasil instigou a presente análise do panorama atual desses produtos e os desafios impostos pelas novas diretrizes.

**MÉTODOS:**

Foi realizado um estudo qualitativo, observacional e descritivo dos suplementos alimentares comercializados em lojas virtuais brasileiras, com o auxílio da ferramenta de busca Google®. Os ingredientes declarados nos rótulos, bem como os efeitos atribuídos a esses produtos e as alegações comerciais utilizadas como forma de promovê-los foram levados em consideração a fim de avaliarmos as mudanças necessárias para o enquadramento legal nas novas diretrizes. Por fim, com o auxílio de base de dados, foram comparados os efeitos declarados pelos fabricantes e atribuídos a determinados ingredientes com as evidências científicas descritas na literatura.

**RESULTADOS:**

No total, foram adquiridos 44 suplementos alimentares provenientes de lojas virtuais brasileiras (n = 7). Das amostras estudadas, 34,2% não poderiam ser enquadradas na categoria Suplementos Alimentares, conforme preconiza a nova regulação da Agência Nacional de Vigilância Sanitária, em decorrência de presença de substâncias não permitidas; 16% dos produtos deveriam ser comercializados como medicamentos. Quanto aos apelos comerciais, 97,7% apresentavam expressões não permitidas. Inúmeras alegações de efeitos atribuídos a determinados produtos, por não possuírem comprovação científica, foram caracterizadas como fraude contra o consumidor.

**CONCLUSÕES:**

Dada a extensa gama de suplementos alimentares e pontos de comercialização, as mudanças necessárias representam um grande desafio regulatório e de produção, esforço este que visa a proteger a saúde dos consumidores. Algumas lacunas previamente existentes ao marco regulatório ainda não foram totalmente solucionadas.

## INTRODUÇÃO

O consumo de suplementos alimentares pela população vem aumentando significativamente nos últimos anos, sendo inúmeras as razões que justificam o fenômeno. A aquisição facilitada em lojas virtuais e a forte influência exercida pelas mídias sociais estão entre os fatores de maior impacto. Entre os anos de 2010 e 2016, no Brasil, houve um aumento no consumo de suplementos alimentares de 233%, chegando a um faturamento de 1,49 bilhão de reais^1^. O elevado número de pessoas consumindo esses produtos por iniciativa própria ou indicação de amigos sem a devida recomendação de profissional especializado tornou-se um problema de saúde pública, preocupando especialistas e autoridades^2^. Há inúmeros relatos na literatura de efeitos adversos indesejáveis, danos hepáticos e até mesmo casos de óbitos ocasionados pela ingestão de suplementos alimentares^3^.

No Brasil, até 2018 não havia uma definição legal para suplementos alimentares. De acordo com a Agência Nacional de Vigilância Sanitária (Anvisa), grande parte dos produtos usados como suplementos alimentares era classificada em diferentes categorias regulatórias: (I) Alimentos para atletas; (II) Suplementos vitamínicos e/ou minerais; (III) Novos alimentos e/ou novos ingredientes; (IV) Alimentos de propriedade funcional e/ou saúde; (V) Medicamentos específicos; e (VI) Fitoterápicos^6^. Existe no mercado um elevado número de produtos, o que dificulta a fiscalização, sendo a falta de segurança para a saúde do consumidor o maior risco associado a esse panorama. Esse quadro favorece a comercialização de suplementos alimentares contendo substâncias proscritas no Brasil, como efedrina e 1,3-dimetilamilamina^12^. Casos de suplementos alimentares adulterados deliberadamente, a fim de intensificar o efeito farmacológico e promover uma falsa imagem de que o produto funciona, são amplamente descritos na literatura^3 , 5 , 13^. Soma-se a isso a escassez de metodologias oficiais no Brasil para a realização desse tipo de análise, tornando duvidosa a qualidade e segurança desses produtos. Fraudes contra o consumidor também foram relacionadas a suplementos alimentares comercializados no Brasil por divergências entre a quantidade de carboidratos e proteínas declarada nos rótulos e os valores reais encontrados após análises realizadas pela Anvisa^16^.

Nos últimos anos, a Anvisa promoveu diversas reuniões a fim de debater e definir uma regulação para os suplementos alimentares. Esses debates originaram a RDC Anvisa nº 243/2018, que define os requisitos sanitários dos suplementos alimentares e caracteriza-se como um marco regulatório no país^17^. A Anvisa também estabeleceu os aditivos alimentares e coadjuvantes de tecnologia autorizados para uso em suplementos alimentares através da RDC Anvisa nº 239, de 26 de julho de 2018^18^. Em contrapartida, os valores de ingestão diária recomendada (IDR) para produtos à base de vitaminas, minerais e aminoácidos deixaram de ser o fator de diferenciação entre suplementos vitamínicos e/ou minerais e medicamentos específicos^7 , 10^. Conforme a nova RDC Anvisa nº 242/2018, serão considerados medicamentos específicos somente os produtos à base de vitaminas e/ou minerais e/ou aminoácidos e/ou proteínas isolados ou associados entre si, para uso oral, com indicações terapêuticas bem estabelecidas e diferentes das alegações utilizadas em suplementos alimentares^19^.

A regularização dos produtos disponíveis no mercado segundo as novas diretrizes legais inclui desde mudanças nas informações contidas nos rótulos até alterações na composição dos suplementos alimentares. O setor regulado tem o prazo de cinco anos para se adequar às novas resoluções.

Este trabalho avaliou as principais não conformidades observadas nos rótulos dos suplementos alimentares e verificou se os efeitos atribuídos a alguns ingredientes tinham respaldo nas evidências científicas. As mudanças requeridas pela nova legislação para os produtos mais populares comercializados no mercado virtual também foram apontadas.

## MÉTODOS

### Aquisição das amostras

Foi realizado um estudo retrospectivo, observacional e descritivo das informações coletadas desde o momento da realização da compra até o recebimento das amostras de suplementos alimentares. As amostras foram adquiridas de janeiro a dezembro de 2017, antes do marco regulatório dos suplementos alimentares, e analisadas conforme as legislações vigentes à época para essa categoria de produtos. Também foram avaliadas as ocorrências de não conformidade e as mudanças requeridas para o enquadramento legal das amostras nas diretrizes atuais. Foram considerados relevantes todos os produtos que apresentavam como indicação “alimentos para atletas” ou “suplemento alimentar para praticantes de atividades físicas”. As amostras foram adquiridas via internet, pesquisadas com o auxílio da ferramenta de busca do Google®. As lojas virtuais (n = 7) eram todas brasileiras, com Cadastro Nacional de Pessoa Jurídica (CNPJ), declaravam o endereço físico nos seus sítios eletrônicos e possuíam serviço de atendimento ao consumidor (SAC). Os preços das amostras variaram de R$ 19,50 a R$ 244,45. De acordo com o orçamento disponível e as características necessárias para o enquadramento do produto como amostra de interesse, foi possível a aquisição de 44 amostras, as quais foram recebidas via postal.

### Classificação das amostras

Os suplementos alimentares foram distribuídos em categorias designadas de acordo com as classificações atribuídas pelos próprios *sites* de venda (suplementos para modulação hormonal, suplementos para perda de peso, suplementos para desempenho sexual e construtores musculares). As informações de rotulagem pertinentes, como ingredientes, lote, fabricante, presença de imagens ou expressões, informações sobre dispensação de registro sanitário conforme a RDC Anvisa nº 27/2010^20^e indicação para o consumo, entre outras, foram tabeladas com o auxílio da ferramenta Microsoft Excel®. Esses dados foram avaliados quanto à conformidade de acordo com a legislação vigente no período de aquisição^6^. Em seguida, as amostras foram analisadas com base nos novos critérios de classificação dos suplementos alimentares, nos ingredientes permitidos e nas alegações dispostas na RDC Anvisa nº 242 e 243/2018^17 , 19^e anexos. As alegações atribuídas aos ingredientes majoritários das amostras foram comparadas com as recomendações de sociedades internacionais e organizações oficiais, como *International Society of Sports Nutrition* ^21^, *Academy of Nutrition and Dietetics, Dietitians of Canada* e *American College of Sports Medicine* ^22^, além de artigos científicos.

## RESULTADOS

Os efeitos atribuídos aos produtos pelos *sites* de venda foram denominados aqui como “apelo comercial”. De acordo com os apelos comerciais, presentes nos sítios eletrônicos, as amostras foram classificadas em quatro categorias principais: (I) Modulador/Precursor hormonal; (II) Perda de peso/Controle de apetite/Diurese; (III) Aumento da libido/Potência sexual; e (IV) Construtor muscular/Melhorador de performance/Ganho de força. A frequência dessas categorias é apresentada no Quadro 1 , bem como os efeitos atribuídos pelos fabricantes como forma de publicidade do produto. Um dos principais pontos observados foi a presença de mais de um ingrediente “ativo” declarado no rótulo de alguns produtos, o que dificultou o enquadramento legal em algumas legislações. No Quadro 1 , estão dispostos somente os ingredientes majoritários, destacando-se a proteína do soro do leite, os ingredientes naturais (espécies vegetais) e as vitaminas e/ou minerais como os de maior frequência.

**Quadro 1:** A frequência entre as categorias dos produtos, efeitos atribuídos aos produtos observados no rótulo e os ingredientes majoritários dos suplementos alimentares estudados.

Categoria % (n)			Efeitos atribuídos aos produtos		Ingredientes principais	

					Descrição	n
I	Modulador/precursor hormonal	29,6 (13)	Precursor/síntese de/ potencializador/aumento da	Testosterona	Zinco, magnésio e vitamina B6^a^	7
					DHEA^b^	2
					*Tribullus terrestris*	2
					Maca peruana	1
					Picolinato de cromo	1
II	Perda de peso/Controle de apetite/diurese	18,2 (8)	Moderador	Apetite	Cafeína	3
					Vitaminas e/ou minerais	3
					*Citrus aurantium*	1
					Psyllium ( *Plantago ovata* )	1
			Melhora/aumenta	Diurese		
			Auxilia/promove	Perda de peso		
III	Aumenta/ reforça e combate/tratamento da potência sexual/libido	13,6 (6)	Aumenta/reforça	Virilidade e libido	*Tribullus terrestris*	2
					Maca peruana	2
					*Eurycoma longifolia*	1
					Zinco, magnésio e vitamina B6	1
			Combate/tratamento da	Impotência sexual		
IV	Auxilia/aumenta/define e promove/proporciona construtor muscular/Melhora da performance/Ganho de força	38,6 (17)	Auxilia/aumenta/define	Massa muscular	Proteína do soro do leite	7
					BCAA^c^	3
					*Paullinia cupana*	2
					Maltodextrina	2
					Zinco, magnésio e vitamina B6	2
					Proteína de carne bovina	1
			Promove/proporciona	Efeitos anabólicos		

		100 (44)			Total	44

^a^Ingrediente declarado no rótulo de duas maneiras: I) com a denominação ZMA; II) como nutrientes adicionados em diferentes proporções.

^b^Deidroepiandrosterona.

^c^Aminoácidos de cadeia ramificada, do inglês *branched-chain amino acids.*

De acordo com os fabricantes, as amostras declaradas como moduladores hormonais (categoria I) (n = 13) eram capazes de aumentar a produção de testosterona ou sugeriam a presença de ingredientes precursores desse hormônio. Seis amostras continham espécies vegetais não permitidas em suplementos alimentares, como o *Tribullus terrestris* e a *Paullinia cupana* , alegando haver propriedades fitoterápicas conforme a RDC da Anvisa n° 26/2014^11^e o Formulário de Fitoterápicos da Farmacopeia Brasileira^23^. Atualmente, de acordo com a instrução normativa (IN) Anvisa nº 28, de 26 de julho de 2018, a *Paullinia cupana* está entre os constituintes autorizados em suplementos alimentares como fonte de cafeína^24^. Ainda nessa categoria, duas amostras continham a deidroepiandrosterona (DHEA), medicamento cujo uso é controlado no país de acordo com a Portaria MS n° 344/1998^25^.

Entre as amostras com alusão à perda de peso (categoria II) (n = 8), todas possuíam apelos comerciais de ação farmacológica, como “moderador de apetite” ou “aumento da diurese”. Um dos produtos que alegava dispensação de registro por se enquadrar como “Alimento para atleta” não poderia ser legalmente classificado nessa categoria, uma vez que declarava no rótulo o *Citrus aurantium,* uma espécie vegetal que classifica o produto em medicamento fitoterápico de ação ansiolítica, sendo a administração recomendada apenas para maiores de 12 anos, o que exige, portanto, o registro junto a Anvisa para sua comercialização^11 , 23^.

Todos os suplementos com indicação de aumento de libido (categoria III) (n = 6) estavam em desacordo com a legislação por atribuírem efeitos terapêuticos aos produtos. Quatro declaravam a presença de espécies vegetais, entre elas o fitoterápico *Citrus aurantium* ^11 , 23^, o *Tribullus terrestris* e a *Eurycoma longifolia* . As duas últimas não se encontram disponíveis no Formulário de Fitoterápicos, mas são passíveis de registro em virtude das alegações terapêuticas apresentadas^26^.

Os suplementos com alegação de construção muscular ou relacionados ao desempenho físico foram os mais frequentes (categoria IV) (n = 17). Dez produtos estavam corretamente classificados como “Alimento para atleta”, considerando os ingredientes declarados nos rótulos, os quais estavam dispensados de registro^6 , 20^. Sete produtos tinham publicidade fazendo alusão ao anabolismo, o que não é permitido em produtos alimentícios^6 , 27^, como suplementos contendo apenas vitaminas e/ou minerais com alegações do tipo: “Gera força e massa muscular” ou “Promove crescimento muscular”. Duas amostras declaravam isenção de registro por se denominarem “Alimentos para atletas”; contudo, em razão da presença de substâncias que não apresentam histórico de consumo no país, como a maca peruana ( *Lepidium meyenii* ), deveriam estar enquadradas na categoria “Novos alimentos e/ou novos ingredientes”^8 , 20^. Ainda conforme a legislação em vigor no momento da aquisição dos suplementos alimentares, duas amostras apresentavam não nutrientes ou fibras alimentares adicionadas, o que não era permitido, conforme inciso IV do artigo nº 8 da RDC Anvisa nº 18/2010^6^. Pelas novas diretrizes, essa adição é possível desde que as substâncias estejam na lista de constituintes autorizados e sejam respeitados os limites mínimos, máximos e as alegações atribuídas^17 , 24^.

Quanto à necessidade de registro das amostras junto à Anvisa, tanto para os produtos nacionais quanto internacionais (n = 4), foi realizada uma análise comparativa entre o que os fabricantes declaravam nos rótulos ( Figura 1A ) e a declaração ideal de acordo com as legislações em vigor no momento da fabricação ( Figura 1B )^20^.

**Figura 1:** Representação gráfica dos registros das amostras avaliadas: (A) declarações pelo fabricante; (B) declarações ideais, conforme as diretrizes regulatórias vigentes.

FDA: Food and Drug Administration.

A Figura 2 detalha a discrepância da diferença entre o enquadramento regulatório dos produtos conforme as informações declaradas no rótulo ( Figura 1A ) e como deveria ser a distribuição entre as categorias após análise dos ingredientes declarados. Dentre as amostras que se declaravam erroneamente isentas de registro, estavam novos alimentos, alimentos com propriedade funcional e até mesmo medicamentos.

**Figura 2:** Distribuição das amostras avaliadas segundo as categorias regulatórias, conforme a legislação vigente no momento da aquisição dos produtos.

De acordo com a nova regulamentação, grande parte dos produtos analisados (70,5%, n = 29) estava em conformidade com a lista de ingredientes permitidos em suplementos alimentares^17 , 24^. Alguns ingredientes que não possuíam, até o momento da aquisição das amostras, o amparo legal para serem adicionados (por exemplo, a *Paullinia cupana* e outros não nutrientes presentes em alimentos para atletas), atualmente, estão autorizados a fazer parte dos constituintes dos suplementos alimentares, respeitando os requisitos necessários^17 , 24^. Por outro lado, quinze amostras (34,2%) não poderiam ser comercializadas como suplementos alimentares por apresentar ingredientes ausentes da lista de constituintes autorizados, como *Camellia sinensis, Lepidium meyenii,* gengibre, canela em pó, *Trichilia catigua* . Quanto ao rótulo, 97,7% das amostras não estavam em conformidade em razão do uso de expressões desautorizadas em suplementos alimentares, que atribuíam propriedades medicamentosas ou terapêuticas ao produto, como aumento da libido e ação diurética^17 , 27^. Além disso, o uso de imagens fazendo alusão a efeitos farmacológicos, como perda de peso ou melhora no desempenho sexual, também foram observados^17^.

## DISCUSSÃO

### Análise dos Rótulos

Até 2018, a denominação “suplemento alimentar” não era prevista por lei e qualquer produto com essa finalidade era comercializado como alimento, em diversas categorias. As amostras estudadas foram adquiridas antes do marco regulatório brasileiro que definiu os suplementos alimentares e que teve como principal objetivo reduzir as fragmentações regulatórias. Até então, o registro sanitário era obrigatório para “Novos alimentos”, “Alimentos com propriedade funcional” e “Alimentos contendo substâncias bioativas ou probióticas”, enquanto os “Alimentos para atletas” e os “Suplementos vitamínicos e/ou minerais” eram isentos de registro^20^.

As não conformidades encontradas nas amostras estudadas foram: (I) ausência de registro de produtos nas categorias “Novos alimentos” (n = 8) e “Alimentos com propriedade funcional” (n = 1); (II) presença de ingredientes que caracterizavam os produtos como medicamentos (n = 7); (III) uso de expressões e alegações indevidas (n = 43).

O DHEA, por exemplo, declarado em duas amostras, não é permitido em suplementos alimentares comercializados no Brasil, por se tratar de medicamento anabolizante precursor de hormônios esteroidais, sujeito a controle especial^25^. Em contrapartida, nos Estados Unidos, esse medicamento é autorizado em suplementos alimentares. Inúmeros efeitos adversos graves, porém, têm sido relacionados ao seu consumo, como taquicardia, arritmias, hepatotoxicidade, entre outros^28^. Ainda, cinco amostras declaravam a presença de espécies vegetais não autorizadas, como *Tribullus terrestres* e *Citrus aurantium* . Entretanto, somente o *Citrus aurantium* apresenta segurança e eficácia baseadas em evidências clínicas, características estas atribuídas à Medicamentos Fitoterápicos^11^. A presença de substâncias não permitidas em suplementos alimentares pode acarretar sérios problemas de saúde pública. A crença do usuário de que se trata de “produto natural” e que, por isso, não trará malefícios à saúde promove um consumo exagerado e, muitas vezes, desnecessário, podendo ocasionar danos à saúde, como hepatotoxicidade, cardiotoxicidade e até mesmo óbito^5 , 29^.

Os produtos comercializados como “Alimentos para atletas” possuíam restrições quanto à composição e adição de outras substâncias, conforme a intenção de uso. Os suplementos proteicos, por exemplo, conforme legislação anterior, não podiam conter fibras e não nutrientes. Os suplementos de cafeína não podiam conter nenhum nutriente ou não nutriente adicionado na formulação^6^. Em razão da legislação vigente no momento da aquisição das amostras, oito apresentaram esse tipo de não conformidade.

No Brasil, não é permitida a adição de medicamentos fitoterápicos em alimentos para atletas^11^. Essa não conformidade foi observada em amostra que continha cafeína associada a *Citrus aurantium* . Altas concentrações de cafeína podem promover efeitos tóxicos, como náuseas, vômitos, arritmias, taquicardia, podendo até mesmo levar a óbito^30^. A sinefrina é um dos compostos ativos do *Citrus aurantium,* frequentemente presente em produtos comercializados para perda de peso. Entretanto, estudos apontam possível cardiotoxicidade relacionada ao consumo indiscriminado^31^. No Canadá, por exemplo, não é permitida a adição de cafeína ou fontes de cafeína em produtos contendo sinefrina por conta do sinergismo de ação, que pode promover efeitos cardiotóxicos, necessitando assim de evidências de segurança comprovadas em humanos^32^.

Essas não conformidades verificadas nos rótulos servem como ferramentas primárias para avaliar a qualidade do produto presente no mercado. Foi perceptível a fragilidade de fiscalização por parte dos órgãos responsáveis, provavelmente pelo grande número de suplementos alimentares disponíveis e de pontos de comercialização, além da facilidade de aquisição permitida pelo comércio virtual. Em decorrência da regulamentação fragmentada e inespecífica vigente durante a aquisição das amostras, ficou evidente a dificuldade de enquadramento legal dos produtos tanto pelos fabricantes quanto pelos comerciantes e consumidores.

### Apelos Comerciais dos Produtos

Os apelos comerciais são ferramentas de publicidade que tem a finalidade de chamar a atenção para possíveis benefícios que a formulação ou algum componente específico promova no organismo. Desde que implementada, a resolução RDC Anvisa nº 259/2002 determina que os alimentos, independentemente da categoria, não podem possuir indicações de propriedades medicinais ou terapêuticas^27^. Expressões que façam referência a anabolismo, catabolismo, hipertrofia, queima de gordura ou aumento da capacidade sexual são vetadas em rótulos, propagandas, panfletos ou outra ferramenta comercial de produtos que foram classificados como alimentos para atletas^6^e que, atualmente, são considerados suplementos alimentares^17^. Essa não conformidade é passível de penalidade, como suspensão da comercialização do produto, multa e até mesmo exclusão dos sítios eletrônicos. No presente estudo, observou-se um número significativo de amostras que apresentavam apelos comerciais e uso de expressões indevidas (97,7%). Sugerem-se as seguintes hipóteses: (I) Elevada quantidade de sítios eletrônicos; (II) Dificuldade de fiscalização de lojas virtuais; (III) A exclusão de um sítio inapropriado não impossibilita a abertura de outro; (IV) Nem sempre são os fabricantes que fazem os apelos comerciais dos produtos, mas, sim, os distribuidores, com a finalidade de aumentar as vendas. O uso de frases que induzam o consumidor à compra pode ser considerada fraude contra o consumidor, já que nem sempre é possível garantir que determinado ingrediente irá proporcionar o resultado esperado.

### Efeitos Atribuídos aos Principais Ingredientes e as suas Evidências Científicas

Inúmeras são as substâncias presentes em suplementos alimentares, mas nem sempre sua presença é justificada em evidências científicas consolidadas. Os efeitos atribuídos aos ingredientes desses produtos muitas vezes não apresentam comprovações fundamentadas ou, até mesmo, não condizem com aqueles já descritos. As lacunas na fiscalização colaboram para o elevado número de produtos que ludibriam o consumidor por seus efeitos milagrosos, levando-o à compra. O marco regulatório surgiu para tentar reduzir essas falhas, através de uma lista de alegações permitidas relacionadas aos constituintes dos suplementos alimentares, disponível no anexo A da Instrução Normativa da Anvisa nº 28, de 26 de julho de 2018^24^. Cabe ressaltar também a frequente presença de ingredientes provenientes de plantas, vitaminas e minerais nas amostras e a imagem de “produto natural” que eles transmitem. O consumidor geralmente acredita que, por ser natural, é seguro e não acarretará efeitos nocivos ao organismo^13 , 14^.

De acordo com Rocha et al. (2016)^3^, a grande procura por esses produtos se dá por três fatores: (I) desconfiança crescente na medicina convencional, com um maior interesse em terapias alternativas; (II) a percepção de que são produtos “naturais”, “saudáveis” e, por se tratar de produtos vegetais, são seguros; e (III) uma tendência crescente da automedicação como forma de aumentar o controle sobre a própria saúde.

A adição de plantas está diretamente relacionada com indicação para reduzir medidas, diurese ou outras alegações que induzem a perda de peso. Contudo, uma espécie pode conter mais de uma substância química e, quando feita de forma incorreta, a extração vegetal pode isolar metabólitos indesejados. Dessa forma, a adição de plantas medicinais deve ser observada cuidadosamente.

O *Tribullus terrestris,* declarado em quatro amostras, é um extrato de planta que tem sido relacionado à estimulação do hormônio luteinizante (LH) e consequente aumento de testosterona^33^. Estudos sugerem que, em decorrência da presença da protodioscina, apresenta uma atividade semelhante a um esteroide. Contudo, não há evidências de que o *Tribullus terrestris* ou algum composto isolado dessa espécie promova mudanças significativas na composição corporal, como ganho de massa magra ou anabolismo^33^.

A cafeína é uma substância natural (1,3,7-trimetilxantina) e consumida em larga escala no mundo inteiro como um estimulante em suplementos alimentares, principalmente aqueles com indicação de perda de peso de forma “natural”. Essa metilxantina é adicionada com o apelo comercial de acelerar o metabolismo, estimular a diurese e promover a queima de gordura. Entretanto, posições oficiais não atribuem à cafeína um aumento na taxa de oxidação de gordura, mas, sim, ao aumento no desempenho do atleta por ser um potente estimulante cerebral^21 , 22^.

Os alimentos proteicos para atletas se popularizaram entre os frequentadores de academias por sua associação com ganho de massa magra. A proteína do soro do leite, popularmente conhecida como *whey protein* , nada mais é do que o sobrenadante obtido da coagulação da caseína, representando de 15 a 20% do leite total. De acordo com a nova regulação, é permitido o uso da alegação de que “as proteínas auxiliam na formação dos músculos e ossos”^24^. Os suplementos de proteína talvez sejam um dos poucos em acordo com as evidências científicas quanto ao ganho de massa magra, já que auxiliam na construção muscular. No entanto, não há relação com níveis de testosterona ou outros efeitos ergogênicos^21 , 22 , 34^, alegações estas encontradas em sete amostras, que utilizaram expressões se referindo ao *whey protein* como capaz de “aumentar níveis hormonais” ou como sendo “um importante precursor da testosterona”.

Os aminoácidos de cadeia ramificada (BCAA), declarados em três amostras, também adquiriram seu espaço dentro das academias. Constituídos de leucina, isoleucina e valina, os BCAA são consumidos com o intuito de reduzir tanto a fadiga física quanto mental, melhorar o desempenho e promover efeitos ergogênicos, como o anabolismo. Os BCAA são responsáveis pela captação de triptofano e sua conversão em serotonina, o qual é responsável pela fadiga muscular^35^. Contudo, os efeitos benéficos desse tipo de suplemento ainda são divergentes na literatura.

No Brasil, os suplementos de BCAA, até o momento da aquisição das amostras, não podiam declarar indicações para atletas ou como alimento para atleta, mesmo sob dispensa de registro^6^. Entretanto, essa não conformidade foi observada em todos os suplementos de BCAA ou contendo BCAA (6,8%), uma vez que foram adquiridos com a especificação de alimento para atleta.

As vitaminas e minerais são suplementos alimentares com a finalidade de complementar as necessidades da dieta de uma pessoa saudável, sem a intenção de substituir algum alimento ou ser usado como dieta exclusiva^7 , 19^. No momento da aquisição das amostras, a regulação da Anvisa considerava que vitaminas e minerais eram alimentos quando contivessem de 25 a 100% da ingestão diária recomendada (IDR) na porção indicada no rótulo^7^. Já se o produto contivesse doses com concentrações equivalentes a mais de 100% da IDR, era considerado medicamento específico^10^. Atualmente, a nova regulação considera vitaminas e minerais como suplementos alimentares, independentemente da dose. Apenas quando apresentarem alegações terapêuticas bem estabelecidas, serão considerados medicamentos específicos, como nos casos do sulfato ferroso, que auxilia nas anemias carenciais, e do ácido fólico, que é indicado para a redução da ocorrência de más formações fetais^19^.

Recentemente, o consumo de suplementos alimentares contendo zinco e magnésio, principalmente entre o sexo masculino, popularizou-se em razão da crença de que essa combinação poderia aumentar os níveis de testosterona e, portanto, promover efeitos anabólicos^36^. Esses minerais são capazes de aumentar as concentrações do hormônio fator de crescimento semelhante à insulina (do inglês *insulin-like growth factor,* IGF-1) em crianças com deficiência; porém, não há evidências comprovadas para essa alegação em pessoas saudáveis^37 , 38^. A combinação de zinco, magnésio e vitamina B6 é também conhecida como ZMA, e alegações de que essa formulação continha substâncias precursoras da testosterona foram encontradas em três amostras. Não há evidências científicas de que esse suplemento auxilie nas adaptações do treino, promova efeitos anabólicos ou promova efeitos significativos nos níveis de testosterona ou no seu metabolismo^21 , 39^.

As atribuições de efeitos milagrosos às substâncias presentes nos suplementos alimentares, ou até mesmo designações de “formulações mágicas”, são recorrentes nesses produtos. Essas alegações nem sempre são atribuídas pelos fabricantes, mas, sim, pelos lojistas como uma forma de publicidade para impulsionar as vendas. Essas atribuições milagrosas também podem ser um indício da presença de substâncias não permitidas em alimentos. O caso do OxyElite Pro®, por exemplo, que foi amplamente divulgado em razão de sua ação na perda de peso, continha 1,3-dimetilamilamina (DMAA), uma substância altamente prejudicial ao organismo. De fevereiro de 2012 a fevereiro de 2014, foram reportados 114 casos ao *Food and Drug Administration* (FDA) de efeitos adversos relacionados ao consumo desse suplemento, caracterizando um grave problema de saúde pública^5 , 40^.

### Regularização dos Produtos Disponíveis no Mercado

A nova legislação para os suplementos alimentares brasileiros busca garantir produtos de qualidade ao consumidor com menos entraves regulatórios para os fabricantes. Os produtos avaliados no presente estudo precisam passar por diversas mudanças para se adequar às novas diretrizes. Essas modificações incluem desde a composição, uma vez que a normativa atual traz uma lista de ingredientes e aditivos permitidos, até a regularização da rotulagem e das alegações utilizadas como forma de promover o produto^17^.

Observou-se elevada frequência no uso de propagandas com alegações sobre determinados ingredientes, nem sempre verdadeiras. Esses apelos comerciais têm o objetivo de induzir o consumidor à compra ou, ainda, promover a falsa imagem de que o produto funciona. Quanto às alegações, de acordo com a nova legislação, das 44 amostras analisadas apenas duas estariam conforme as normas previstas no anexo V, da Instrução Normativa nº 28, de 26 de julho de 2018^17 , 24^. As normas de propagandas são menos obedecidas por fabricantes e distribuidores em decorrência da dificuldade de fiscalização pela agência reguladora. Muitas vezes, é mais vantajoso aos fabricantes pagar as multas decorrentes ao uso de alegações apelativas, uma vez que estas impulsionam as vendas e a popularização do produto. O atual sistema de regulatório para os suplementos alimentares objetivou reduzir essas não conformidades por meio da relação de constituintes autorizados, seus limites mínimos e máximos e alegações permitidas. No entanto, as novas diretrizes ainda não são ferramentas facilitadoras tanto para as indústrias quanto para o consumidor, uma vez que há alguns balizadores. Por exemplo, caberá ao fabricante definir as quantidades adequadas a serem ingeridas nas recomendações diárias de consumo do produto e por grupo populacional em casos em que os limites mínimos e máximos não estiverem estabelecidos^17^.

O crescimento no número de suplementos alimentares disponíveis no mercado, bem como o elevado número de pontos de comercialização, colabora para a ineficiência na fiscalização desses produtos. Cabe salientar que a facilidade de aquisição via comércio virtual pode fazer o consumidor ter acesso a produtos ilegais ou que contenham substâncias não autorizadas em suplementos alimentares. O usuário nem sempre está ciente dos danos que essas substâncias podem causar, configurando-se um desafio à saúde pública. Políticas de educação que mostrem os r'iscos associados ao consumo desnecessário, alertas que auxiliem a reconhecer a qualidade dos suplementos alimentares e o incentivo à busca de profissional capacitado antes da aquisição de qualquer produto dessa natureza são boas estratégias para conscientizar a população e, consequentemente, reduzir o comércio de produtos ilegais ou em desacordo com a legislação.
